# Patients’ use and views of real‐time feedback technology in general practice

**DOI:** 10.1111/hex.12469

**Published:** 2016-04-28

**Authors:** Christine Wright, Antoinette Davey, Natasha Elmore, Mary Carter, Luke Mounce, Ed Wilson, Jenni Burt, Martin Roland, John Campbell

**Affiliations:** ^1^ Primary Care Research GroupUniversity of Exeter Medical SchoolExeterDevonUK; ^2^ The Primary Care UnitInstitute of Public HealthUniversity of CambridgeCambridgeUK

**Keywords:** patient feedback, primary care, real time feedback, survey

## Abstract

**Background:**

There is growing interest in real‐time feedback (RTF), which involves collecting and summarizing information about patient experience at the point of care with the aim of informing service improvement.

**Objective:**

To investigate the feasibility and acceptability of RTF in UK general practice.

**Design:**

Exploratory randomized trial.

**Setting/Participants:**

Ten general practices in south‐west England and Cambridgeshire. All patients attending surgeries were eligible to provide RTF.

**Intervention:**

Touch screens were installed in waiting areas for 12 weeks with practice staff responsible for encouraging patients to provide RTF. All practices received fortnightly feedback summaries. Four teams attended a facilitated reflection session.

**Outcomes:**

RTF ‘response rates’ among consulting patients were estimated, and the representativeness of touch screen users were assessed. The frequency of staff–patient interactions about RTF (direct observation) and patient views of RTF (exit survey) were summarized. Associated costs were collated.

**Results:**

About 2.5% consulting patients provided RTF (range 0.7–8.0% across practices), representing a mean of 194 responses per practice. Patients aged above 65 were under‐represented among touch screen users. Receptionists rarely encouraged RTF but, when this did occur, 60% patients participated. Patients were largely positive about RTF but identified some barriers. Costs per practice for the twelve‐week period ranged from £1125 (unfacilitated team‐level feedback) to £1887 (facilitated team ± practitioner‐level feedback). The main cost was the provision of touch screens.

**Conclusions:**

Response rates for RTF were lower than those of other survey modes, although the numbers of patients providing feedback to each practice were comparable to those achieved in the English national GP patient survey. More patients might engage with RTF if the opportunity were consistently highlighted to them.

## Introduction

There is increasing focus in the UK NHS on improving the quality of services and in collecting information from service users on their experience of care.^1^ A recent review^2^ summarizes a range of strategies for measuring patient experience: each has strengths and limitations, and their appropriateness depends in part on the aim of gathering feedback. Strategies such as focus groups, panels and patient stories provide rich data, but have limited generalizability. Surveys typically represent a less personalized but more generalizable and pragmatic approach to collecting information about patients’ experiences and are currently the principal method used in the UK National Health Service (NHS).

Historically, the main vehicle for collecting patient feedback in the NHS has been the National Patient Survey Programme, including the GP patient survey, although other methods (such as local surveys, patient comments and formal complaints) may be used by practices to identify areas for potential service improvement. For example, the Friends and Family Test is a single question approach promoted within the NHS which seeks to provide patients with the opportunity to feedback on their care.^3^ Patient surveys have been criticized because of concerns about their administrative burden and cost, the usefulness of information collected^4^ and the lack of timeliness and regularity of feedback. Patients who may not have recent experience of their general practice could be surveyed, and patients with particularly strong views may be more motivated to respond.^4^, ^5^

There is growing interest in the use of new technologies to collect patient experience data, because, in theory, this enables results to be assessed and acted on quickly.^6^ Real‐time feedback (RTF) involves the systematic collection, analysis and reporting of information from individuals who have recently used a service. The approach typically employs touch screen kiosks or hand‐held devices available at the point of service use,^6^, ^7^, ^8^ but paper‐based approaches (such as postcards) have also been used.^4^, ^6^

To date, there has been little published research focusing on the use of RTF in UK general practice despite this setting representing the lynchpin in access to health care in the UK. A six‐month pilot study conducted in 22 UK general practices concluded that RTF could be implemented successfully in most practices and could be used for performance improvement in this setting.^9^ It was, however, noted that the process needed to be actively promoted to fully engage practice staff and patients. While other literature^1^, ^6^, ^7^ has highlighted the need to monitor participation rates and the characteristics of patients who provide RTF, the UK pilot study in general practice^9^ did not report this information.

Studies of RTF in primary care clinics in the USA^7^, ^10^ have reported completion rates of 40–50% when clinic staff actively direct patients to touch screens and encourage them to provide feedback after a consultation. One study^10^ noted that older people and minority ethnic groups were less likely to use the technology, but reported that the process did not adversely affect waiting times or other aspects of the practice routine. However, under present staffing arrangements in the UK NHS, allocating staff to exclusively support RTF is unlikely to prove feasible in routine practice.

Collection of patient feedback is insufficient on its own to improve services.^11^ Best practice guidance^1^, ^6^, ^8^ recommends that organizations should also reflect and act on feedback while that feedback remains fresh. A recent review^12^ suggests that much is known about how to collect patient experience information, but that less is known about how organizations can use this to effect service change and improve patient experience.

One potential mechanism for stimulating change is external facilitation.^13^ Facilitated feedback is recommended by the UK General Medical Council for individual doctors who undertake patient and colleague surveys in the context of medical appraisal and revalidation. As part of their appraisal process, doctors must reflect on their multisource feedback results, discuss these with a trained facilitator (such as their appraiser) and use the feedback to inform their future professional development plan.^14^ In other settings, a UK pilot study has reported that ward‐level feedback meetings with a researcher were more likely to stimulate improvements in nursing care than written patient survey results alone.^15^

### Study aims and objectives

This study aimed to investigate, in a small number of UK general practices, the feasibility and acceptability of a real‐time feedback intervention, which might have the potential to inform service improvement in general practice. Our study focussed on practices with low communication scores as this is a subject area where feedback is common, but commonly not acted on,^12^ which is of importance – with poor communication accounting for a substantial proportion of referrals in GMC complaints processes,^16^, ^17^ and since communication is, furthermore, an area which is potentially amenable to change.^18^

Specifically, the objectives were to: (i) pilot potential RTF interventions consisting of RTF collection and reporting, with or without facilitated feedback; (ii) estimate the proportion of consulting patients who use RTF touch screens in practice waiting areas; (iii) describe the characteristics of patients who use RTF touch screens to provide feedback; (iv) observe the extent to which practice staff encourage patients to use touch screens; (v) elicit patient views on touch screens as a means of leaving feedback; and (vi) estimate the costs associated with undertaking and delivering RTF.

## Method

### Design

The research had two phases: a feasibility study (January to June 2014) and an exploratory trial (July 2014 to February 2015). The feasibility study findings (not reported here) were used to refine the RTF intervention and methods for the exploratory trial. In summary, the feasibility study which was carried out in two practices highlighted the potential for low response rates, the limited engagement of busy staff with the RTF process, the ambivalence of patients towards RTF and challenges in taking action in response to patient feedback.

In the exploratory trial, practices were randomly assigned to one of four RTF intervention groups (4 groups × 2 practices = 8 practices) or to a control group (2 practices). Randomization occurred in two blocks (each of five practices) using a simple randomization approach based on random number generation. Randomization was conducted by a statistician, otherwise unconnected to the project and blind to practices’ identity. Stratification by variables such as practice size or GP patient survey score was not attempted due to the small number of practices involved.

RTF interventions varied on two dimensions: the provision of a team reflection session (facilitated or unfacilitated feedback) and the level of feedback reporting (team only or team plus individual clinician). The four intervention combinations were therefore:

- facilitated team‐level feedback (Group A);
- facilitated team‐ and individual‐level feedback (Group B);
- unfacilitated team‐level feedback (Group C); and
- unfacilitated team‐ and individual‐level feedback (Group D).

Practices in the control group did not collect RTF during the intervention period but had the use of two touch screens and received team‐ and individual‐level feedback reports (but no facilitation) after the trial.

### Setting and participants

General practices in the south‐west of England and in Cambridgeshire were eligible to take part in the exploratory trial if they fell in the lowest 50% of scores on the communication items in the previous year's GP patient survey (2013 data). Eligible practice teams were invited to take part in a number of recruitment ‘waves’ until the target of 10 practices was reached. To facilitate fieldwork, practices within reasonable travelling distance of the Universities of Exeter and Cambridge were prioritized in the sampling frame.

Initial study information packs were posted to practices and followed up by a telephone call from the local researcher. Detailed briefing sessions were organized at practices that expressed an interest in participation. Practice managers or research leads (GPs or nurses) provided written consent on behalf of the practice team.

### Intervention

#### RTF collection

Two touch screens were installed in the waiting areas of participating practices – typically there was one free‐standing kiosk and one desktop device. Practice staff attended a short, interactive training session covering the day‐to‐day management of the touch screen devices. Installation of hardware was supported by Customer Research Technology (CRT) Limited. After an initial ‘run‐in’ period of up to 1 week, live RTF collection continued for twelve consecutive weeks.

Practice staff were responsible for encouraging patients to use the touch screens to provide feedback about their experience at the practice that day. As we wished to assess the potential of RTF in routine practice, additional support for the RTF collection process was not provided. Publicity materials were provided in waiting rooms to encourage patient participation. These publicity materials included postcards for clinicians to hand to consulting patients, a large pull‐up poster and leaflets in the waiting area. Practice teams were also encouraged to publicize the touch screens via their websites, newsletters or information display screens.

During the RTF collection period, patients who consulted a health professional or visited the practice for other reasons (for example, to book an appointment or collect a prescription) were eligible to provide feedback. Feedback could not be collected from patients who received a home visit or consulted a clinician by telephone.

The survey was initiated and navigated by touching the screen and could be completed by the patient or their proxy (for example a parent/guardian, relative or carer). Due to limited funding, survey items were presented in English only.

A series of questions and response options (Table 1) were displayed on the touch screen, including the NHS Friends and Family Test,^3^ nine items focusing on access, communication and satisfaction derived from the GP patient survey,^19^ two practice‐tailored questions (on issues relevant to the practice's own interest) and basic demographic items (patient's age, gender and ethnic group). To reduce the length of the survey, only four items used in the GP patient survey to assess communication skills were included. Based on previous research,^19^ the three items loading most strongly, plus one item loading least strongly, onto overall communication scores for GPs and nurses were selected. Filter questions were included to ensure that respondents were presented only with items that were relevant to their visit – for example, patients who had not had a consultation were not asked to rate the skills of a health professional. The final screen invited free‐text comments.

**Table 1:** Summary of RTF survey items and response options

Question Source/Type	Wording of item	Response options presented
NHS Friends and Family Test	How likely are you to recommend our GP surgery to friends and family?	Extremely likely/Likely/Neither likely nor unlikely/Unlikely/Extremely unlikely/Don't know
GP patient survey	How easy is it to get through on the telephone to this practice?	Very easy/Fairly easy/Not very easy/Not at all easy/Haven't tried or Don't know
GP patient survey	How easy is it to get an appointment for a time that suits you?	Very easy/Fairly easy/Not very easy/Not at all easy/Haven't tried or Don't know
GP patient survey	How helpful do you find the receptionists at this GP surgery or health centre?	Very helpful/Fairly helpful/Not very helpful/Not at all helpful/Don't know
GP patient survey	Overall, how satisfied are you with the care you get at this GP surgery or health centre?	Very satisfied/Fairly satisfied/Neither satisfied nor dissatisfied/Fairly dissatisfied/Very dissatisfied
Filter question	Have you had an appointment with a health professional at the practice today?	Yes/No
Filter question	*If ‘Yes’*: Which of the following health professionals did you see?	Doctor/Nurse/Health‐care assistant or Phlebotomist (for a blood test)/Practice counsellor/Other health professional
Filter question	Which doctor or nurse did you see today?	List (and photographs) of individual staff at the practice plus: Another doctor/Another nurse/Don't know
GP patient survey	Do you have confidence and trust in the doctor or nurse you saw today?	Yes, definitely/Yes, to some extent/No, not at all/Don't know or Can't say
GP patient survey	How good was the health professional at each of the following (a) Giving you enough time (b) Listening to you (c) Treating you with care and concern (d) Taking your problems seriously	Very good/Good/Neither good nor poor/Poor/Very poor/Doesn't apply
Practice specific items	Up to two items (with relevant response options) on topics selected by the practice team were included after the clinician communication skills items, or (for patients who had not consulted a health professional) after the overall experience item.	
Respondent information	Are you …	The patient/Parent or guardian of the patient/Spouse or partner of the patient/Another relative or friend of the patient/Other
Patient's gender^a^	Are you/Is the patient …?	Male/Female
Patient's age group^a^	How old are you/How old is the patient?	Below 18/18–25 years/26–45 years/46–65 years/Above 65 years
Patient's ethnic group^a^	What is your ethnic group/What is the patient's ethnic group?	White/Mixed/Asian or Asian British/Black or Black British/Chinese or Other
Free‐text comments	If you would like to leave any further comments, please type below	Space for free‐text comments

a Where the respondent was the patient, subsequent demographic items (gender, age group and ethnic group) were phrased as ‘Are you’ … Where the respondent was someone other than the patient (‘proxy’), the demographic items were phrased, ‘Is the patient …’.

#### Feedback reporting

Patient feedback was transmitted from the touch screens to CRT Limited via Wi‐fi or 3G connections. Where no reliable signal was available, researchers manually downloaded data approximately once a fortnight.

Feedback was not included in practice reports generated by CRT Limited under certain circumstances, for example if response options were selected in a time frame that suggested the responder could not have read the questions, or if practice staff typed an agreed code in the comment box to indicate they had been demonstrating the touch screen. Otherwise, feedback was regarded as valid and was included in the practice reports. Free‐text comments were screened by the local researcher and details that might identify individual patients removed. Negative comments about a clinician's practice or standards of care were discussed with the chief investigator on an individual basis to determine a course of action proportionate to the risk to patients. This process was invoked on only two occasions. We encouraged practices to nominate one member of staff to have oversight of the RTF data collection and staff engagement.

Practice teams received up to six feedback reports (approximately fortnightly), which summarized their cumulative patient feedback in the form of frequency tables and graphs. In half of the exploratory trial practices (Group B or D), practitioners were provided with individualized reports where at least 20 patients had identified in their RTF responses that they had consulted the named practitioner. Team‐level reports were e‐mailed to the practice manager for dissemination to the wider team, while personalized reports were e‐mailed or posted direct to the individual clinician.

#### Feedback facilitation

Practice teams allocated to exploratory trial groups A and B were offered a session with an experienced facilitator (a GP tutor or appraiser) which lasted 45–60 min. Facilitation, where this was undertaken, took place on only one occasion in each of the practices, and this took place either halfway through or towards the end of RTF period. During the session, clinical and non‐clinical members of the team were encouraged to reflect on the practice's patient feedback, to identify the practice's strengths and weaknesses and to discuss whether any changes to the service might be indicated. The practice team was also encouraged to develop a ‘SMART’^20^ plan for initiating change.

### Main outcomes

#### Patients’ use of touch screens

To calculate an ‘RTF rate’ and explore the representativeness of patients who used the touch screens, information was extracted from two sources. There was no reliable method of ascertaining the number of patients who attended the surgeries for reasons other than a consultation, and therefore, the RTF rate calculation was restricted to consulting patients.

Firstly, anonymized data were extracted from each practice's appointments system to determine (a) the number of appointments attended with any health professional during the 12‐week RTF collection period and (b) the characteristics (age/gender) of consulting patients. As practices do not routinely record patient ethnicity, we were unable to incorporate this variable in our consideration of participation rates.

Secondly, anonymized information about patients who provided valid RTF during the same time period was extracted from datasets provided by CRT Limited. This included the age and gender of consulting patients and the type of health professionals (GP, nurse, health‐care assistant or other) they had consulted.

The two sources of data described above were used to calculate the proportion of consulting patients who used the touch screens (overall and for each practice) using the following equation:

$$\begin{array}{cc} & \text{Number of patients who provided valid RTF and reported having a consultation}\\ & \frac{\;\;\;\text{with a health professional}\;\left(\text{ascertained from each}\;{\text{practice}}^{\prime }\text{s final RTF dataset}\right)}{\;\;\text{Number of patients who consulted a health professional in the same}}\\ & \text{period}\;\left(\text{ascertained from each}\;{\text{practice}}^{\prime }\text{s computerized appointments system}\right) \end{array}$$

To identify whether particular patient groups were more likely to use the touch screens than others, the proportions of patients who provided valid RTF across different age and gender groups were compared with the respective proportions of all patients who consulted in the same time period (ascertained from the appointments search) using *z*‐tests.

Not all patients who provided RTF reported their age or gender. As the proportion of missing data was small (reported below), missing data were excluded.

#### Patients’ views and experiences of RTF

To further assess the feasibility and acceptability of RTF collection, researchers regularly visited the eight intervention group practices and spent time in waiting areas. Practice visits occurred approximately once a fortnight and were organized on different weekdays and at a range of times to capture workload and staffing variations and a range of practice activity.

During each practice visit, researchers observed patient and staff interactions in relation to the touch screens (over a two‐hour period) and conducted exit surveys (over a 1 hour period) of a convenience sample of patients to elicit their views about RTF, whether or not they had used a touch screen. A poster was displayed in the waiting area during visits to explain the researcher's presence.

For observational work, individual patient and staff consent were not sought in case this significantly altered behaviour relating to the touch screens.^21^ For 1 hour of each observation session, a structured checklist^22^ was used to systematically record (using Yes/No tick boxes) the frequency of a range of pre‐specified interactions between patients and practice staff and patients’ use of the touch screens and publicity materials during the observation periods. The checklist had been piloted during the feasibility study to ensure its content was comprehensive and relevant. Data were recorded anonymously to protect patient and staff confidentiality. Interactions were summarized descriptively. This included, for example, the number (%) of patients who interacted (for any reason) with practice staff in the waiting area and the number (%) patients who were encouraged by reception staff to use the touch screen.

For the exit surveys, patients gave verbal consent to participate and responded verbally to a series of structured questions about their use/non‐use of the touch screens. Patients who had used a touch screen were asked about their experience of this. Patients who had not used a touch screen were asked about their reasons for not doing so. All participants were asked about their general views of RTF as a means of collecting patient feedback. Brief demographic information (age/gender group) was recorded. Patient responses were summarized descriptively, that is the number (%) percentage of patients endorsing each response option.

#### Cost analysis

This analysis sought to estimate the cost of providing an RTF intervention in GP practices over the 12‐week period of live RTF collection.

Costs were estimated from the perspective of the NHS and included the cost of the following: renting and installing the touch screen devices, professionally printed publicity materials, generating fortnightly feedback reports, staff attendance at set‐up training, and staff and facilitator attendance at the facilitation sessions. Cost data for the hire of touch screens and provision of team‐level and individual‐level reports were provided in aggregate by the RTF provider. Time inputs for practice staff and facilitators were collated for each of the practices based on attendance lists for the set‐up training and facilitation sessions. Unit costs for staff were extracted from standard UK sources.^23^, ^24^

The price year for analysis was 2014 and costs included value added tax (VAT) where applicable. Given the exploratory nature of the study and the small sample size, summary costs only were reported and no attempt was made to draw comparisons between exploratory trial groups.

## Results

### Practice recruitment and characteristics

In the exploratory trial, eight practices were recruited from Devon, Bristol and North Somerset, and two were recruited from Cambridgeshire. The characteristics of participating practices are summarized in Table 2. Nine practices had pre‐existing touch screen appointment check‐in arrangements in place although none had touch screens that enabled patients to comment on their care.

**Table 2:** Characteristics of participating general practices

	Group A		Group B		Group C		Group D		Controls	
	Practice 1	Practice 2	Practice 3	Practice 4	Practice 5	Practice 6	Practice 7	Practice 8	Practice 9	Practice 10
List size^a^^,^ b	4114	4568	3618	8005	13 000	15 189	10 998	9500	11 727	6675
Number of staff^b^										
GPs	3	4	3	6	11	6	12	–	6	4
Nurses	2	3	1	5	3	7	7	–	8	2
Health‐care assistants	2	1	1	1	3	2	2	–	2	1
Reception/admin	6	8	7	12	12	12	17	–	16	8
Managerial	1	3	2	2	3	2	3	–	2	1
Setting^b^	Rural	Urban	Urban	Inner city	Rural	Urban	Urban/rural	Inner city	Urban	Urban
GP patient survey centile score^c^	33.4%	39.5%	28.9%	14.3%	34.0%	31.9%	21.9%	32.7%	27.1%	14.5%
Deprivation decile^d^	8	2	10	2	6	2	9	7	7	7
Mean consultations per week	441.6	707.0	181.3	620.3	1809.5	474.6	434.0	636.8	1040.2	250.3

a Average list size for England is 7041.

b List size, staff mix and setting data was provided by the practice at the start of the exploratory trial. Practice 8 did not provide numbers of staff employed.

c Derived from the practice's overall scores on communication items in the national GP patient survey (Year 7 data).

d Deprivation data^26^; lower numbers indicate more deprivation.

### Patients’ use of touch screens

During the RTF collection period, a total of 1941 of 79 145 (2.5%) consulting patients provided valid feedback, representing a mean of 194 valid responses per practice. The mean response rate in the practices was 3.2% (SD 2.2%; range 0.7–8.0%). Table 3 presents response by practice, and by patient gender and age, using data from seven of the ten practices (appointments data could not be broken down by these demographics for three practices). For these seven practices, the mean percentage of RTF responders who did not provide their gender was 6.7% (range 1.9–13.7%) and 6.7% (range 2.2–13.7%) did not provide their age.

**Table 3:** Post‐consultation real‐time feedback response rates

	Responses/Appointments	% (95% CI)
Overall	1941/79 145	2.5 (2.3–2.6)
Practice (trial group)^a^		
1 (Intervention A)	231/5299	4.4 (3.8–4.9)
2 (Intervention A)	201/8484	2.4 (2.1–2.7)
3 (Intervention B)	110/2175	5.1 (4.2–6.1)
4 (Intervention B)	168/7443	2.3 (1.9–2.6)
5 (Intervention C)	162/21 764	0.7 (0.6–0.9)
6 (Intervention C)	64/5695	1.1 (0.9–1.4)
7 (Intervention D)	416/5208	8.0 (7.0–8.8)
8 (Intervention D)	102/7642	1.3 (1.1–1.6)
9 (Control)	386/12 482	3.1 (2.8–3.4)
10 (Control)	101/3003	3.3 (2.7–4.1)
Gender^b^		
Men	501/23 739	2.1 (1.9–2.3)
Women	816/34 226	2.4 (2.2–2.6)
Age band^a^		
Below 18s	139/6747	2.1 (1.7–2.4)
18‐25s	72/3998	1.8 (1.4–2.3)
26‐45s	298/12 383	2.4 (2.1–2.7)
46‐65s	450/15 190	3.0 (2.7–3.2)
Above 65s	357/19 647	1.8 (1.6–2.0)

a Intervention A, Facilitated reflection; practice‐level feedback; B, facilitated reflection, practice‐level plus practitioner‐level feedback; C, No facilitated reflection, practice‐level feedback; D, No facilitated reflection, practice‐level plus practitioner‐level feedback.

b Appointments data could not be broken down by gender or age for three out of ten practices. As such, the numbers displayed do not sum to the overall totals given.

### Representativeness of consulting patients who provide RTF

The age and gender of consulting patients who provided RTF at the 7 of 10 practices for which appointments data could be broken down by age and gender are summarized in Table 4, together with the characteristics of all patients from these practices who consulted during the study period. We observed a slightly higher proportion of females in the RTF responders (62.0%) than in the consulting population (59.0%), *P *=* *0.034. The proportion of RTF responders aged under 18 years or in the 26‐ to 45‐year‐old age bands did not differ significantly from the proportions in the consulting population. However, there were significantly less responders in the 18‐ to 25‐year‐old (5.5% of responders; 6.9% of the population, *P *=* *0.043) and the 65 years or older (27.1% of responders; 33.8% of the population, *P *<* *0.001) age bands and significantly more responders aged 46–65 years old (34.2% of responders; 26.2% of the population, *P *<* *0.001).

**Table 4:** Representativeness of post‐consultation responders to real‐time feedback

Characteristic, n (%)	Responders out of total (%)	Proportion in population (%)	*P*‐value
Women^a^	816/1317 (62.0)	34 226/57 965 (59.0)	0.034
Age band^a^			
Below 18s	139/1316 (10.6)	6747/57 965 (11.6)	0.228
18‐25s	72/1316 (5.5)	3998/57 965 (6.9)	0.043
26‐45s	298/1316 (22.6)	12 383/57 965 (21.4)	0.262
46‐65s	450/1316 (34.2)	15 190/57 965 (26.2)	<0.001
Above 65s	357/1316 (27.1)	19 647/57 965 (33.8)	<0.001
Ethnicity^b^			
White	1724/1941 (88.8)	n/a	–
Mixed	28/1941 (1.4)	n/a	–
Asian	52/1941 (2.7)	n/a	–
Black	27/1941 (1.4)	n/a	–
Chinese	8/1941 (0.4)	n/a	–
Missing	102/1941 (5.2)	n/a	–

a Appointments data could not be broken down by gender or age for 3 out of 10 practices. These proportions are taken from the real‐time feedback and appointments data of the seven contributing practices.

b Appointments data could not be broken down by ethnicity for any practice.

### Observed patient and staff interactions

Researchers conducted six structured observation sessions at each of the eight intervention group practices in the exploratory trial. No observations were conducted at the control group practices.

During these sessions, 873 of 1205 (72.5%) attending patients were observed by the researcher to have some form of verbal interaction with a receptionist in the waiting area. In a much smaller number of observations (0.8%), patients interacted with a health professional in the waiting area. Where staff–patient interactions were observed, only 60 of 1199 (5%) patients were encouraged to use the touch screens by a receptionist, and no health professionals were observed to draw the devices to the attention of patients.

When patients were encouraged by receptionists to use the touch screen, 36 of 60 (60%) patients actually attempted to start the survey. In contrast, only 28 of 1114 (2.5%) patients attempted the survey without direct encouragement. Few patients (78 of 1199; 6.5%) were observed to read publicity materials in the waiting area.

### Patient views and experiences of RTF

Across all eight intervention group practices, 375 patients participated in the exit surveys. Of these, 103 (27.5%) patients had used a touch screen in the waiting area.

The majority of patients who reported using a touch screen (87 of 101; 86.1%) had positive views of RTF as a way of leaving feedback for the practice team (data missing for two exit survey participants). All reported that they had found it easy to complete the RTF survey and that they answered all questions. Most responders (79 of 98; 80.6%) reported completing the survey in 2 min or less (data missing for five participants).

Over half of the patients who had not provided RTF (149 of 268; 55.6%) were unaware of the touch screens or the opportunity to leave feedback. Patients who were aware of the touch screens gave a number of other reasons for not using them: 29 of 84 (34.5%) reported they did not have time to leave feedback; 15 of 84 (17.9%) had concerns over using the technology; 12 of 84 (14.3%) reported providing RTF on previous visits and did not know they could leave feedback more than once; 5 of 84 (6.0%) felt their feedback would not be relevant (e.g. because it was positive); and 4 of 84 (4.8%) had concerns over anonymity or how feedback would be used. Although they had not used the touch screens during their current visit, 178 of 260 (68.5%) patients were positive about the idea of providing RTF in this way.

### Cost analysis

The costs associated with RTF interventions A to D are summarized in Table 5 and comprised RTF equipment hire, practice staff training (all four interventions) and facilitated feedback sessions (A and B only). Total costs ranged from £1125 (for unfacilitated/team‐level feedback) to £1887 (for facilitated/team ± individual‐level feedback).

**Table 5:** Analysis of cost of RTF in general practices

Item	Intervention Group:	A	B	C	D	All groups mean (standard deviation)	Groups
	Feedback level^a^:	T	T&I	T	T&I		
	Facilitated session?	Yes	Yes	No	No		
	Number of practices	2	2	2	2	8	
RTF equipment – hire and provision							
Publicity (posters & leaflets)	750 postcards + one pull‐up poster per practice	£107					
Touch screen (kiosk) rental	12 week hire (total)	£630					
Touch screen (desktop) rental	12 week hire (total)	£342					
Kiosk collection	‐	£38					
Reporting^b^	‐	£75					
Total						£1117	(A–D)
Practice staff set‐up session		£43	£34	£8	£22	£27 (£22)	(A–D)
Total						£1144 (£22)	(A–D)
Facilitated reflection session							
Facilitator fees		£250	£250	N/A	N/A	£250 (£58)	(A, B)
Practice staff to attend session		£477	£378	N/A	N/A	£428 (£180)	(A, B)
Total		£727	£628			£678 (£227)	(A, B)
Total cost		£1887	£1779	£1125	£1139		

a T: Team‐level reports provided; T&I: Team‐ and individual‐level reports provided.

b Cost of reporting was averaged over all eight intervention group practices. The marginal cost of individual‐level feedback over group‐level feedback was assumed to be zero. An hourly rate of £109.00 was assumed for GPs, £51.00 for nurse practitioners, £34.00 for practice nurses; £10.06 for health‐care assistants, £21.54 for practice managers, £10.78 for administrators and £9.35 for receptionists.^23^, ^24^

Costs associated with the hire of touch screens, provision of fortnightly feedback reports and provision of publicity materials were common to all practices. In this study, such costs were covered from the research grant. Over the twelve weeks, these costs totalled £1117 per practice. The largest component was the rental of two touch screens (total £972 per practice).

Staff time for attending set‐up training was assumed to be 15 min per team member and was estimated at £27 per practice (SD £22). Practice managers and administrative staff attended the training in most practices; GPs, nurses and health‐care assistants tended only to attend in practices allocated to a facilitated feedback intervention (groups A and B).

Facilitated feedback sessions (for groups A and B only) cost an estimated £678 per practice (SD £227), comprising £250 (SD £58) in fees to the facilitator and £428 (SD £180) for practice staff time to attend facilitation.

## Discussion

Real‐time feedback (RTF) is a relatively novel method for collecting information about patient experience in UK general practice. In this study, patients generally reported positive views about the idea of RTF as a means of providing feedback to practice teams. However, actual engagement with the touch screens in the waiting area of participating practices was lower than that reported in studies from the USA.^7^, ^10^

In absolute terms, the majority of practices in the current study collected feedback from 100 or more patients, which compares favourably to the volume of feedback achieved by the same practices in the most recently published national GP patient survey.^25^ However, the proportion of consulting patients who used the touch screens in this study varied across practices (range 0.7–8.0%) and, overall, feedback represented the views of a relatively small proportion (mean 3.2%) of consulting patients. Response rates for the same practices in the national GP patient survey, drawing on a postal survey of registered patients, were much higher (range 27–53%).^25^ The low response rates seen in this research may be one contributing factor in the apparent reluctance of practices to engage meaningfully with the feedback, for example by instituting changes in service provision, or investing effort in discussing the results within the practice.^4^, ^13^ Ensuring higher response rates may be one important approach in addressing this apparent inertia.

The difference in response rates between the current study and the studies in US primary care clinics may reflect a lower level of direct encouragement and support by practice staff to facilitate patients’ use of the touch screens. At most practices, receptionists were given responsibility for encouraging patients to provide RTF, rather than clinicians. However, the researchers’ observations were limited only to waiting areas and some clinicians may have highlighted the opportunity for patients to provide feedback when they were in the consultation room.

In the waiting areas, receptionists were observed to interact with a significant proportion of patients who attended the surgery but they were rarely observed to encourage the use of the touch screens. While a number of reasons were given by patients in the exit surveys for not using the touch screens, over half of those patients had been unaware of the opportunity to leave feedback; others may have provided feedback if clearer information had been provided about the presence and purpose of the touch screens. Where reception staff did encourage the use of touch screens, patients were more likely to start the survey. Direct encouragement appeared to be much more effective than publicity materials displayed in the waiting area.

In our study, response rates were broadly similar for males and females, with female patients only slightly more likely to provide RTF than males (2.4% versus 2.1%, respectively). This is in line with the most recently published national GP patient survey data, where approximately even proportions of males (49%) and females (51%) responded.^25^ The observation of lower rates of feedback in older age groups is in line with that reported by Dirocco and Day^10^ where more intensive staff support with RTF had been available. That study also reported lower feedback rates among ethnic minority groups. Our study was unable to investigate this important variable as appointments data could not be categorized by patient ethnicity at any of the participating practices.

We estimated the average cost of providing RTF (including staff training) at £1144 per practice for the twelve‐week period. Given a mean of 194 responses per practice, this yields a cost of approximately £5.90 per response. This may seem excessive, however, the cost of RTF needs to be compared with outcomes to judge whether RTF represents a good investment for a GP practice or for the health service. The ideal outcome would be an improvement in patient benefit or experience resulting from the practice team's response to the feedback, but this is outside the scope of this exploratory trial. Outside the context of a research project, the costs of hiring touch screens may be borne directly by the practice or service, alongside staff time invested in set‐up training and team meetings to reflect on patient feedback. Interestingly, GPs and nurses tended only to attend set‐up training sessions in practices allocated to facilitated feedback, suggesting clinician engagement may have been higher in those practices. This may be worthy of more detailed investigation in future studies, as it could be a mediator of any observed outcomes.

To maximize the touch screen usage of patients, consistent effort and time from practice staff is needed to directly encourage and support feedback from patients. This could be seen as time well spent if it resulted in the collection of RTF from a larger number of patients who are representative of the patient population who actually use practice services.

### Strengths and limitations

A range of general practices were recruited to the study, including those in urban, inner city and rural settings, with varying deprivation scores and list sizes. However, practices were drawn from only two broad geographical areas (south‐west England and Cambridgeshire), which may not be representative of the UK as a whole. Furthermore, practices were recruited from those in the lower half of practices based on communication item scores in the previous national GP patient survey. Practices who agreed to participate in the exploratory trial may have been those with an interest in research or service improvement and may not be representative of other practices who fall in the lower range of GP patient survey scores.

The collection of RTF in this study has a number of limitations when compared to other means of obtaining feedback. For example, RTF survey items were presented only in English, and patients who did not visit the surgery during the study period were unable to provide their feedback. In some practices, it proved difficult to extract demographic information about consulting patients from the practice system and there was some evidence that appointments data were not consistently recorded within systems, limiting reliable assessment of the response rate and the representativeness of patients who used touch screens compared to the consulting population. In this study, it was not possible to calculate response rates for patients who attended the surgery for reasons other than a consultation.

The collection and reporting of RTF in each practice took place over one 12‐week period. This may have been too long a period for practices to invest sustained staff effort and time in encouraging patient feedback amid other demands inherent in routine UK general practice. In addition, the pilot nature of the study precluded long‐term follow up, for example in respect of whether changes in service provision had been considered, introduced and sustained. Future studies should consider the optimum time period for collecting RTF in general practice, perhaps favouring a more intensive effort to collect feedback for a shorter period of time with the process being repeated after a suitable interval to enable assessment of the impact of any resulting service change on patient experience.

## Conclusions

Despite the low RTF response rate achieved when touch screens were located in UK general practice waiting areas, patients were broadly positive about the concept of real‐time feedback and found the touch screens easy to use. More intensive and consistent support and encouragement from practice staff might increase the overall number of patients who use touch screens and ensure feedback is collected from a more representative sample of patients who use a practice's services. To improve staff engagement with the process, future studies should involve practices in the design and content of RTF surveys. To maximize patient participation, future studies should also seek to address language barriers and patient concerns about the use of technology. Shorter ‘bursts’ of RTF collection and reporting may be more acceptable to and sustainable for practices and would allow a more thorough assessment of the degree to which RTF can be collected and used to improve patient experience in general practice settings.

## Funding

This work was funded by a National Institute for Health Research (NIHR) Programme Grant for Applied Research (RP‐PG‐0608‐10050). The NIHR played no part in designing the study, in the collection, analysis and interpretation of the data, in the writing of the article, or in the decision to submit it for publication. All researchers in the study are independent of the funding body.

## Study approvals

An NHS ethics application was submitted to the National Research Ethics Service (NRES) London – Riverside Committee (Reference: 14/LO/0015) in November 2013. After initial screening of the application, the Research Ethics Committee decided the project met criteria for ‘audit/service evaluation’ (rather than ‘research’) and therefore did not require review by an NHS Research Ethics Committee. The NRES decision was supported by the study Sponsor (Royal Devon and Exeter NHS Foundation Trust). The project underwent research governance reviews by Research and Development Offices in each of the participating NHS sites, and relevant approvals were obtained before fieldwork commenced.
